# Focus group on conflict management in the classroom in Secondary Education in Costa Rica: mixed methods approach

**DOI:** 10.3389/fpsyg.2024.1407433

**Published:** 2024-10-03

**Authors:** Pedro Bonilla R., Immaculada Armadans, M. Teresa Anguera

**Affiliations:** ^1^Vice-Rectorate of Research, Universidad Estatal a Distancia, San José, Costa Rica; ^2^Department of Social Psychology and Quantitative Psychology, Faculty of Psychology, PsicoSAO, Research Group in Social, Environmental and Organizational Psychology, Institute of Research in Education, University of Barcelona, Barcelona, Spain; ^3^Faculty of Psychology, Institute of Neurosciences, University of Barcelona, Barcelona, Spain

**Keywords:** focus group, observational methodology, observation instrument, mixed methods, conflict management, behavioral relationships

## Abstract

**Introduction:**

The educational system in Costa Rica, as regulated by Law #7727, which governs Alternative Conflict Resolution and Promotion of Social Peace must consider the interplay of various factors influencing classroom conflicts and the management strategies employed by teachers. Consequently, it becomes imperative to identify the most effective conflict resolution practices applicable within this context. To achieve this, a thorough procedure based on mixed methods was employed to analyze and interpret both classroom conflict behaviors and teachers’ strategic responses.

**Method:**

This study employed an indirect observational methodology, from a mixed methods approach. Data was collected through a focus group comprised of teachers. Following the connect framework, the procedure was conducted in three phases: QUAL-QUAN-QUAL. Two analytical techniques were implemented. A lag sequential analysis was used to explore potential behavioral patterns. The results of this analysis informed a polar coordinate analysis, which generated a visual representation of the relationships between codes.

**Results and discussion:**

A focus group addressed four questions, yielding satisfactory data quality control results (kappa values: 0.80, 0.77, 0.76, 0.82). In polar coordinate analysis, the 37 observational instrument categories were designated as focal behaviors. Each of the 37 analyses treated all categories as conditioned behaviors. The analysis identified 342 activation/inhibition relationships between focal and conditioned behaviors. Of these, 195 were statistically highly significant, distributed across quadrants: Quadrant I (106), Quadrant II (36), Quadrant III (16), Quadrant IV (36). Significant gaps in understanding of conflict were identified, along with disparities in the resources and competencies necessary for successful conflict resolution among the observed educators. Specifically, teachers frequently employed techniques informed by intuition rather than deliberate strategy learned in their training. Consequently, the study advocates for enhancing teachers’ cognitive and emotional competencies to optimize conflict management within the classroom and bolster their adaptive coping mechanisms.

## 1 Introduction

Costa Rican education faces a deficit in general training and updating, including in the fields of conflict resolution and violence prevention training, for both students and educators (Estado de la Nación, 2023). This stems, in part, from prioritizing the acquisition of functional competencies for specific labor demands, sometimes at the expense of communication skills and values essential for healthy coexistence.

Considering Law #7727 (Procuraduría General de la República (PGR), 2004), which regulates actions for Alternative Dispute Resolution and the Promotion of Social Peace in Costa Rica, Article 1 states that,

Every person has the right to adequate education on peace, in schools and colleges, which have the duty to make their students understand the nature and demands of the ongoing construction of peace… The Higher Education Council will seek to include, in official educational programs, elements that promote the use of dialogue, negotiation, mediation, conciliation, and other similar mechanisms as suitable methods for conflict resolution (p. 1).

Probably the lack of training programs in skills and strategies for conflict management within educational institutions is a contributing factor to the emergence of violence cases in that environment.

Ministerio de Educación Pública (2023) reveals a concerning trend. In the first month of 2022, 30 violent incidents were reported in educational institutions. This number escalated significantly in the first month of 2023, with 77 cases of violence and 24 cases of bullying.

To drive transformative change in educational conflict management, Pineda-Alfonso and García-Pérez (2017) emphasize the necessity of re-examining our understanding of conflict and coexistence. They assert that…

… There is a great deal of parallelism between the causes and consequences of individual and social conflicts, and there is confusion or overlap between personal and social concerns; It is often the case that social interests are conceived only in terms of future personal success (p. 151).

Research demonstrates the essential role teachers have in mediating conflict within classrooms. Pantoja (2005) advocates that educators should have the skillset to shape student behavior, promoting conflict resolution as a naturally acquired competency.

On the other hand, Villalta (2014) underscores the necessity to examine contributing factors to the emergence of conflict, which encompass institutional structures, teacher proficiency, and the overall educational climate.

Addressing deficiencies in both the theoretical and practical understanding of conflict resolution among educators is crucial. A robust body of research on individual, group, and societal influences on conflict exists (Galli, 2004; Luhmann, 2007; Martucelli, 2014; Pineda-Alfonso and García-Pérez, 2017; Redorta, 2019; Stewart and Barrick, 2000).

Furthermore, the Integrated Circular Model of Conflict (Bonilla et al., 2020) facilitates the analysis of the individual, group or social aspects that influence conflicts within educational institutions, supporting the development of essential social, emotional, and cognitive aspects to improve coexistence spaces.

Taking as reference Costa Rican Social Security Fund (2021) data reveals workplace stress as the primary cause of disability among Costa Rican educators in 2019, with a marked rise in neurotic disorders driven by anxiety and depression.

This study’s objective was to pinpoint behavioral patterns and the qualitative aspects fueling classroom conflict and the strategies employed by Costa Rican secondary school teachers for their resolution.

A mixed-methods approach, incorporating a focus group to gain insight into teachers’ experiences, was utilized. Analysis of indirect observations provided valuable results with the potential to inform future research aimed at enhancing classroom conflict management.

## 2 Methods

### 2.1 Mixed methods approach

This study is situated within the framework of mixed methods. While the first authors to mention the term mixed methods were Parkhurst et al. (1972), Newman and Benz (1998), more than a quarter of a century ago, set out to explore the qualitative-quantitative interactive continuum in research (Onwuegbuzie and Hitchcock, 2015; Onwuegbuzie and Johnson, 2021), considering the classical dichotomy that both options respond to opposing paradigms as false and unfounded. In this direction, there are no pure quantitative methods that do not involve qualitative elements in some stages of the process (Chang et al., 2009; Nzabonimpa, 2018; Sandelowski, 2014). The development of mixed methods has been exponential (Anguera, 2022), and it is still in the process of maturation, given the multiple existing methodological sensitivities. However, the current focus is on the integration of qualitative and quantitative elements (Anguera et al., 2017; O'Cathain et al., 2010; Onwuegbuzie, 2012).

We take as a reference the following words of Creswell and Plano Clark (2011, p. 7): “There are three ways in which mixing occurs: merging or converging the two datasets by actually bringing them together, connecting the two datasets by having one build on the other, or embedding one dataset within the other so that one type of data provides a supportive role for the other dataset” (p. 7) [emphasis added]. Taking this quote as a reference, we initiated a rethinking of quantitizing, which is a crucial element of mixed methods. “Connecting the two datasets by having one build on the other” means that a qualitative database can be transformed into another qualitative database while maintaining its informative quality but modifying its appearance to make it more organized.

Inspired by this notion, we focus on the re-elaboration and transformation of qualitative data, a process known as quantitizing. This involves preserving the essential information within a qualitative dataset while restructuring it for enhanced organization.

Structuring mixed-methods research effectively necessitates a comprehensive framework. To meet this need and based on the via *connect* approach proposed by Creswell and Plano Clark (2011), we employ the QUAL-QUAN-QUAL macro-stages (Anguera et al., 2020). This approach represents both a conceptual and procedural advancement in mixed-methods research. By applying this process to the focus group, which is entirely innovative, in the first qualitative stage (QUAL), qualitative data are obtained, a customized observation instrument is built, the text is segmented into units, and appropriately coded; in the QUAN stage, data quality control is carried out, and quantitative analysis is performed; finally, in the second QUAL stage, the results are interpreted. This establishes a bridge between qualitative and quantitative research paradigms.

This study employs indirect observation methodology, gathering data from teachers in a focus group setting. This method is particularly suitable for analyzing classroom conflict and resources for managing conflict, such as emotional regulation and coping mechanisms. Indirect observation is highly valuable in behavioral research as it enables textual analysis of verbal behavior or documents. In this study, it offers a robust framework for observing, systematizing, and analyzing teacher responses within the focus group (Anguera et al., 2018).

### 2.2 Design

This research aims to study classroom conflicts and the resources teachers utilize for their management, including emotional regulation and coping strategies. Data were obtained from educators participating in a focus group. This approach aligns with Anguera et al. (2018), who advocate for the value of indirect observation value in analyzing everyday behaviors.

Indirect observation analyzes textual material, including verbal transcripts or existing documents. Here, this technique facilitated rigorous observation and systematization of teacher narratives obtained from the focus group.

This study applies observational methodology, specifically indirect observation, always within the framework of mixed methods. In observational studies, there are eight possible designs (Sánchez-Algarra and Anguera, 2013), resulting from combining three dichotomous criteria: units (individual vs. multiple), temporality (single session vs. intersessional following), and dimensionality (single dimension vs. some dimensions). This study employed a nomothetic, punctual, and multidimensional (N/P/M) design. This denotes the involvement of multiple participants (secondary school teachers), data collection within a single session with intrasessional follow-up (following from the beginning to the end of the session), and the existence of some dimensions derived from the study’s theoretical framework.

### 2.3 Focus group and participant profile

In this research project, focus groups were selected as the qualitative data collection method. This approach facilitates the open expression of thoughts and ideas in a conducive group setting (Flynn et al., 2018), providing in-depth insights into teacher reasoning and behaviors (Thofehrn et al., 2013).

The inclusion criteria for selecting the 14 participating teachers in the two focus groups were: (1) Teachers with experience levels spanning 2 to 28 years were purposefully recruited. (2) Their professional backgrounds encompassed varied teaching contexts, including rural, urban, urban–rural, and coastal settings. (3) Seven men and seven women were selected. (4) Teachers who demonstrated willingness and commitment to participate in the two previously scheduled work sessions were chosen.

Teachers with less than 2 years of professional experience were excluded, as well as those who had only experienced urban or rural schools, and those who did not demonstrate commitment to participate in the research.

For participant recruitment, permission was obtained through the offices of the Directors of the two schools where the focus groups took place. A meeting was held with 3 groups of 10 teachers each, with the objective of explaining the research, its scope, and limitations, and to determine their availability to participate in the study.

From there, those who voluntarily decided to participate were asked to sign an informed consent form in which they agreed to be part of the research under strict confidentiality, both regarding their identities and the information they provided.

Both the focus groups and the 8 interviews conducted were moderated and administered by one of the researchers involved in the preparation of this article, and they were conducted in a context of high complexity, considering the enforcement of law #9999 in August 2021 (Asamblea Legislativa, 2021).

### 2.4 Observation instrument

An *ad hoc* observation instrument was meticulously built to indirectly assess the verbal behaviors of participating teachers. Its design was grounded in both the theoretical framework and emergent themes from the focus group data. Employing a hybrid format combining field notes and category systems (Anguera et al., 2007), the instrument is outlined in Table 1.

**Table 1:** Direct observation instrument created *ad hoc* for this study.

Dimension	Sub-dimension	Category systems	Codification
**1. Typology of the conflict** (Coie and Dodge, 1998; Moore, 1995; Muñoz et al., 2007; Underwood et al., 2001)	A. Interpersonal	Verbal Aggression	1A1AV
		Physical Assault	1A2AF
		Irritation	1A3I
		Irrational Communication	1A4ICR
		Repetitive Negative Behaviors	1A5CNR
	B. Structural	Insufficient Coping Resources	1B1DIRAC
		Inter-institutional Disarticulation	1B2FAI
		Power Relations	1B3RP
	C. Interests	Contrasting Objectives	1C1CO
		Disinterest in the other Party in Conflict	1C2NCIOP
		Discourse of the Parties to a Conflict	1C3DPC
		Incompatible Needs	1C4NI
	D. Value	Imposing Beliefs on the other Person	1D1ICOP
		Disregard for the other’s Opinion	1D2NCOOP
	E. Information	Poor Information to Make Decisions	1E1EITD
		Different Interpretation of Information	1E2DII
**2. Aspects that affect the emergence of conflict** (Abbagnano, 1986; Anguera, 1999; Arciniega et al., 2008; Feldman, 1999; Merleau-Ponty, 1975; Owji et al., 2013; Simmel, 1908; Vollmer, 1974)	A. Individuals	Perception of the Conflicting Event	2A1PEC
		Perceptible Behavior in the Natural Environment	2A2CPEN
	B. Groups	Lack of Trust	2B1FC
		Lack of Identity with the Group	2B2FIG
	C. Social	Lack of Social Skills	2C1FHS
		Associative Behavior Tendency	2C2TCA
		Dissociative Behavior Tendency	2C3TCD
**3. Coping strategies** Dual interest model (Blake and Mouton, 1964; Hall, 1969; Pruitt and Rubin, 1986; Rahim and Bonoma, 1979; Thomas, 1976)	A. Integration	Mutually Friendly Solution	3A1PSFAPC
	B. Domination	Satisfaction of Self-Interest	3B1SIP
	C. Servility	Assignment of One of the Parties	3C1UPCO
	D. Avoidance	Low Intensity to Negotiate	3D1BIPGN
	E. Commitment	Intermediate Solution to the Conflict	3E1APBSI
**4. Teaching skills to manage conflict** (Alves and Pinto da Costa, 2021; Garaigordóbil and Martínez-Valderrey, 2016; Ibarrola-García et al., 2017; Lithoxoidou et al., 2021; Puertas-Molero et al., 2018; Valente and Lourenço, 2020; Zee et al., 2017)	A. Social	Empathy with the other Party in Conflict	4A1EPC
		Prosocial Behavior	4A2CPS
	B. Emotional Issues for Conflict Negotiation	[Auto] Regulates emotions	4B1RE
		Perspective-taking on the Conflict	4B2TPC
		Perception of Effectiveness in Dealing with Conflict	4B3DPEAC
		Awareness of the Conflict Situation	4B4FCSC
	C. Cognitive	Conflict Analysis/Critical Thinking	4C1APCC
	D. Communication	Active Listening	4D1EA
		Proper Tone of Voice	4D2UTVA
		Clear Information	4D3CI

This study used a combination of field format and category systems to be advantageous. The category system ensured theoretical alignment, while the field format provided adaptability. This instrument modality reduced the limitations of the category system, which is very rigid, and the field format, which lacks theoretical consistency.

The indirect observation instrument comprises four dimensions: (1) Typology of Conflict, (2) Aspects Influencing Conflict Emergence, (3) Coping Strategies, and (4) Pedagogical Skills for Conflict Management. These dimensions were further refined into sub-dimensions, each with an exhaustive and mutually exclusive system of categories. In total, 38 distinct categories were employed, each assigned a unique code.

### 2.5 Procedure

These focus groups studies explored the factors influencing classroom conflict and the conflict resolution skills possessed by teachers. Participants were asked:

1. What are the primary factors contributing to classroom conflict? Please consider individual, group, and social aspects (e.g., lack of empathy for others’ positions, lack of group trust, individual disengagement from the group, inadequate social skills, etc.).
2. What skills do you possess to address classroom conflict (e.g., cognitive, emotional, communication, negotiation, reflection, analytical skills, perspective-taking)? Which of these were emphasized during your teacher training?

Focus group dynamics adhered to the guidelines outlined by Hamui-Sutton and Varela-Ruiz (2013). The moderator encouraged open dialogue and equitable participation, fostering a natural conversational flow.

Verbal responses were transcribed and analyzed indirectly. Textual units were segmented and coded, with close attention paid to transcription accuracy, segmentation, and the development of code matrix.

Transcriptions are vital for preserving focus group data. Following Bohle (2013), best practices were employed: (a) careful selection of transcribed content with relevant contextual information, (b) segmentation of text into meaningful units, (c) structuring the text according to participant turns, and (d) incorporation of codes.

Segmenting the transcribed data was complex due to the overlap of different dimensions/sub-dimensions. To resolve this, a taxonomy was used (Anguera, 2021), adhering to Krippendorff's (2013) criteria: (1) orthographic (punctuation-based), (2) syntactic (grammatical sentences), (3) contextual (shifts in topic), and (4) interlocutory (speaker changes). The combined interlocutory-syntactic approach was optimal for this study.

During this stage of the process, quatitizing occurred using a mixed methods approach. To do this, we obtained the parameters of frequency and order or sequence.

Following Anguera et al. (2018), 233 textual units were identified. A mixed-methods approach was used for analysis, obtaining frequency and order/sequence data (Bakeman, 1978). Sequential order was essential for code matrix construction, aligning with Schegloff’s (2007, p. 9) definition of sequence as “a course of action implemented through talk.”

The record was materialized through code matrices, where the columns represent the subdimensions of the observation instrument and the rows represent the successive textual units. The cells in a row correspond to the different co-occurrences of that textual unit. The coding was done manually using the Excel program.

### 2.6 Data quality control

Prior to data analysis, the coded observational data matrices must undergo rigorous quality control. This essential step in observational methodology mitigates potential biases inherent in the coding process, a risk that is heightened when using indirect observation techniques. To ensure reliability, a minimum of three records are required for agreement calculations. The Kappa coefficient, introduced by Cohen (1968), was computed using the free GSEQ software^1^ (Bakeman and Quera, 2011).

This study employed an intra-observer agreement analysis at three distinct time points, with a minimum one-week interval between assessments. Using indirect observation, three successive records were performed on 15% (approximately 35 textual units each) of the transcriptions derived from both focus group questions. A minimum one-week separation was maintained between records. Partial kappa coefficients were calculated independently for each question and subsequently averaged to determine intra-observer agreement. Table 2 presents these results.

**Table 2:** Kappa coefficients and the assessment of intra-observer agreement for each question.

Kappa coefficients					
Questions	Encodings	Encodings	Encodings	Average	Assessment
	1–2	1–3	2–3		
1	0.72	0.92	0.67	0.77	Enough
2	0.67	1.0	0.80	0.82	Almost Perfect

The rating is indicated according to the criteria established by Landis and Koch (1977): 0.01–0.20 Sligth; 0.21–0.40 Fair; 0.41–0.61 Moderate; 0.61–0.80 Substantial; 0.81–1.00 Almost perfect.

Following the completion of data quality control, comprehensive data analysis was undertaken.

### 2.7 Data analysis

The present study employed a mixed-methods approach integrating qualitative and quantitative elements. Within the QUAL-QUAN-QUAL process’s second macro-state, lag sequential analysis was performed first, followed by polar coordinate analysis.

Lag sequential analysis (Bakeman, 1978) constitutes a first stage of the polar coordinate analysis. Its purpose was to identify regularities (behavioral patterns) from categorical data records (qualitative data).

The lag sequential analysis allows for the detection of behavior patterns, following the guidelines of Bakeman (1978), and the results it provides are the adjusted residuals resulting from contrasting unconditional and conditional probabilities based on one or several given behaviors and one or several conditioned behaviors, after applying the correction of Allison and Liker (1982). This analysis can be carried out prospectively (forward, with positive lags) or retrospectively (backward, with negative lags). Results are obtained from adjusted residuals. This technique has broad applicability, including focus groups, in-depth interviews, and other text-based sources.

The adjusted residuals enable the second step of the QUAN stage: polar coordinate analysis (Sackett, 1980). This technique maps interrelationships between codes within an observational record and represents these relationships as vectors. Analysis requires a prior record containing sequential code occurrences/co-occurrences. A focal behavior is selected based on the research objective, with other categories serving as conditioned behaviors. This study employed a modified concept of retrospectivity (Anguera, 1997) for enhanced effectiveness (Gorospe and Anguera, 2000; Hernández-Mendo et al., 2012; Tarragó et al., 2017).

**Figure 1:**
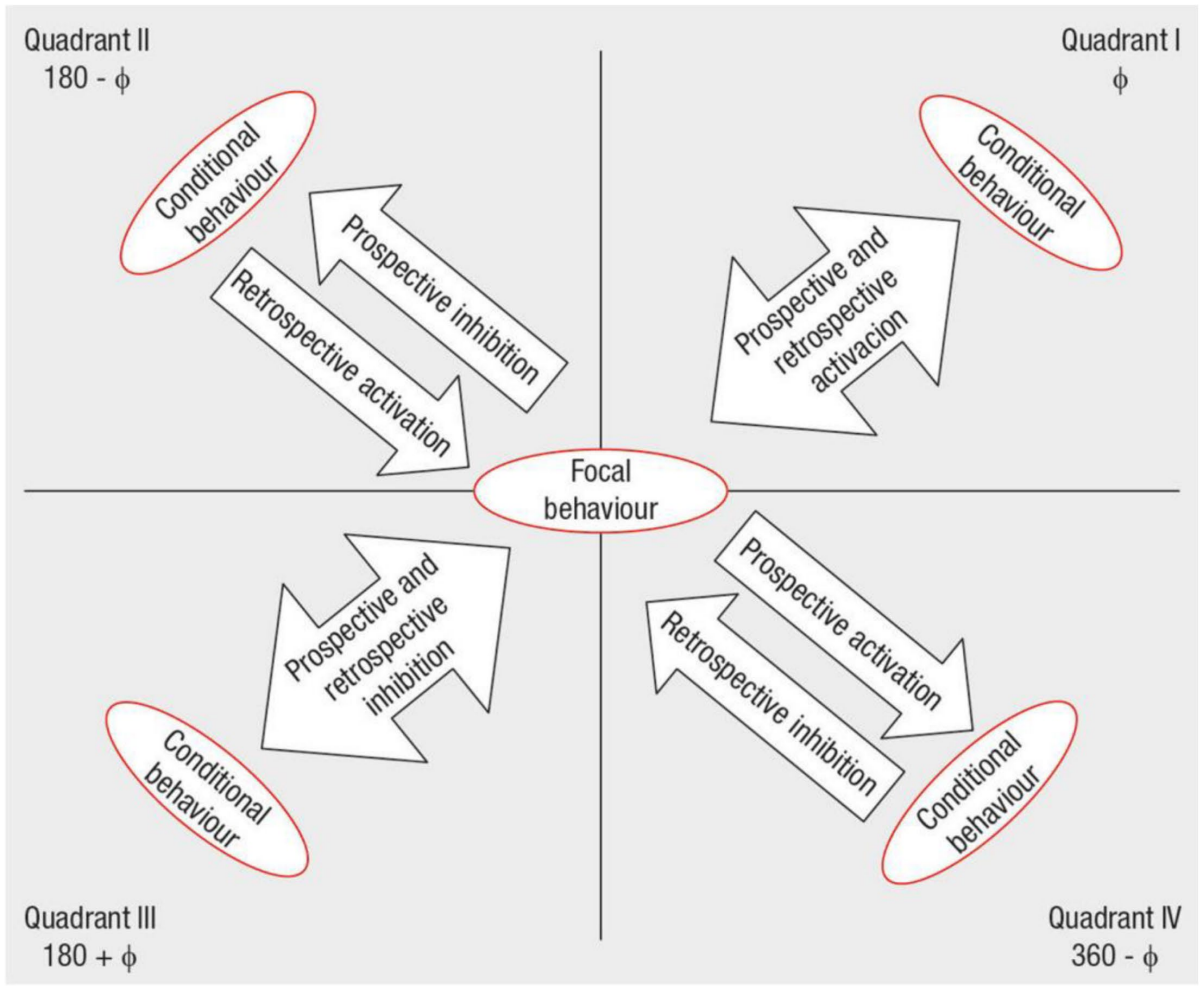
Interpretation of the relationships between focal behavior and conditioned behaviors in each. Excerpted from Aragón et al. (2017, p. 30) with permission of the authors.

After obtaining adjusted residuals from lag sequential analysis, prospective and retrospective Zsum parameters (Cochran, 1954) are calculated for each conditioned behavior across an equal number of positive and negative lags. This step significantly reduces data complexity (Anguera and Losada, 1999). Vector length and angle are then determined, facilitating graphical representation of the relationship between focal and conditioned behaviors. Vector angle (and thus its quadrant) reveals the nature of this relationship (Figure 1), while length indicates statistical significance.

Polar coordinate analysis is conducted using HOISAN software (Hernández-Mendo et al., 2012). The free program R (Rodríguez-Medina et al., 2022) has been used to optimize vector visualization graphically.

## 3 Results

The data analysis aimed to identify behavioral patterns via lag sequential analysis and to map inter-category relationships using polar coordinate analysis.

Due to the extensive results, a detailed commentary focuses on the first category within the first sub-dimension of each of the four dimensions: Dimension 1 (Typology of Conflict), Sub-dimension A (Interpersonal): *Verbal Aggression* (1A1AV). Dimension 2 (Aspects that Affect the Emergence of Conflict), Sub-dimension A (Individuals): *Perception of the Conflictive Event* (2A1PEC). Dimension 3 (Coping Strategies), Sub-dimension A (Integration): *Mutually Friendly Solution* (3A1PSFAPC). Dimension 4 (Teaching Skills to Manage Conflict), Sub-dimension A (Social): *Empathy with the Other Party in Conflict* (4A1EPC).

As outlined in Section 2.7, lag sequential analysis serves to detect behavioral patterns. While primarily instrumental in this study, as the adjusted residuals will form the basis for polar coordinate analysis, it will also provide complementary insights that inform the interpretation of both analyses.

**Dimension 1: Focal behavior *Verbal Aggression***

Supplementary Table S1 presents adjusted residuals of the lag sequential analysis (considering 1A1AV as the given behavior and all categories as conditional). Prospective lags (+1 to +5) and retrospective lags (−1 to −5) were assessed.

Supplementary Table S1 emphasizes adjusted residuals exceeding 1.96 (significance level of 0.05), which form the basis for identifying behavioral patterns. Interpretation follows conventional rules (Anguera et al., 2021), specifically focusing on the second conventional rule: a behavioral pattern ends when two consecutive lags exhibit empty conditional probabilities (i.e., probabilities less than or equal to the unconditioned probability).

The prospective patterns from given behavior 1A1AV are:

**1A1AV →** 1A1AV **→**: *Verbal Aggression* then activates another *Verbal Aggression.*
**1A1AV →** 1A2AF: *Verbal Aggression* activates *Physical Aggression.*
**1A1AV →** 1A4ICR **→** 1A41CR: *Verbal Aggression* activates *Irrational Communication*, which is maintained.
**1A1AV →** Empty lag **→** 1C3DPC: *Verbal Aggression,* although not immediately, activates the *Discourse of the parties to a conflict.*
**1A1AV →** Empty lag **→** 1E1EITD: *Verbal Aggression,* although not immediately, activates *Poor Information to Make Decisions*.
**1A1AV →** Empty lag 1E2DII **→** 1E2DII **→**: *Verbal Aggression,* though not immediately, activates *Different Interpretation of Information*, which is maintained.

The retrospective patterns before given behavior 1A1AV are:

1A1AV ← **1A1AV:** *Verbal Aggression* is usually preceded by *Verbal Aggression.*
1A4ICR ← 1A4ICR ← **1A1AV:** *Verbal Aggression* is usually preceded by *Irrational Communication*, which is persistent.
1D2NCOOP ← 1D2NCOOP ← Empty lag **→ 1A1AV**: *Verbal Aggression*, though not immediately, is preceded by a D*isregard for the Other’s Opinion*, which is persistent.
1B3RP ← **1A1AV:** *Verbal Aggression* is preceded by *Power Relations.*
2B2FIG ← **1A1AV:** *Verbal aggression* is preceded by L*ack of Identity with the Group.*
1C2NCIOP ← 1C2NCIOP ← Empty lag ← **1A1AV:** *Verbal Aggression*, though not immediately, is preceded by *Disinterest in the other Party in Conflict*, which is persistent.
1E2DII ← Empty lag ← **1A1AV:** *Verbal Aggression*, though not immediately, is preceded by D*ifferent interpretation of Information*.

The parameters of the polar coordinate analysis have been calculated from the adjusted residuals, which are shown in Table 3.

**Table 3:** Values of the parameters corresponding to the analysis of polar coordinate, considering 1A1AV as focal behavior, and all categories as conditioned behaviors.

Category	Quadrant	Prospective Zsum	Retrospective Zsum	Length	Signification	Angle
1A_1A2AF	IV	2.04	−0.75	2.18	*	339.82
1A_1A4ICR	I	2.46	2.46	3.48	**	45
1B_1B1DIRAC	II	−1.94	0.77	2.09	*	158.47
1C_1C3DPC	I	1.22	3.01	3.24	**	67.9
1C_1C4NI	I	3.01	0.45	3.04	**	8.53
1D_1D2NCOOP	II	−0.58	6.59	6.62	**	95.02
1E_1E2DII	I	2.45	0.89	2.61	**	20

Only conditioned behaviors in which significant and very significant values have been obtained are included.

Table 3, in addition to the prospective Zsum and retrospective Zsum values, shows the values corresponding to the length and angle of the vector in all cases where they are significant (length > 1.96, for a significance level of 0.05).

**Figure 2:**
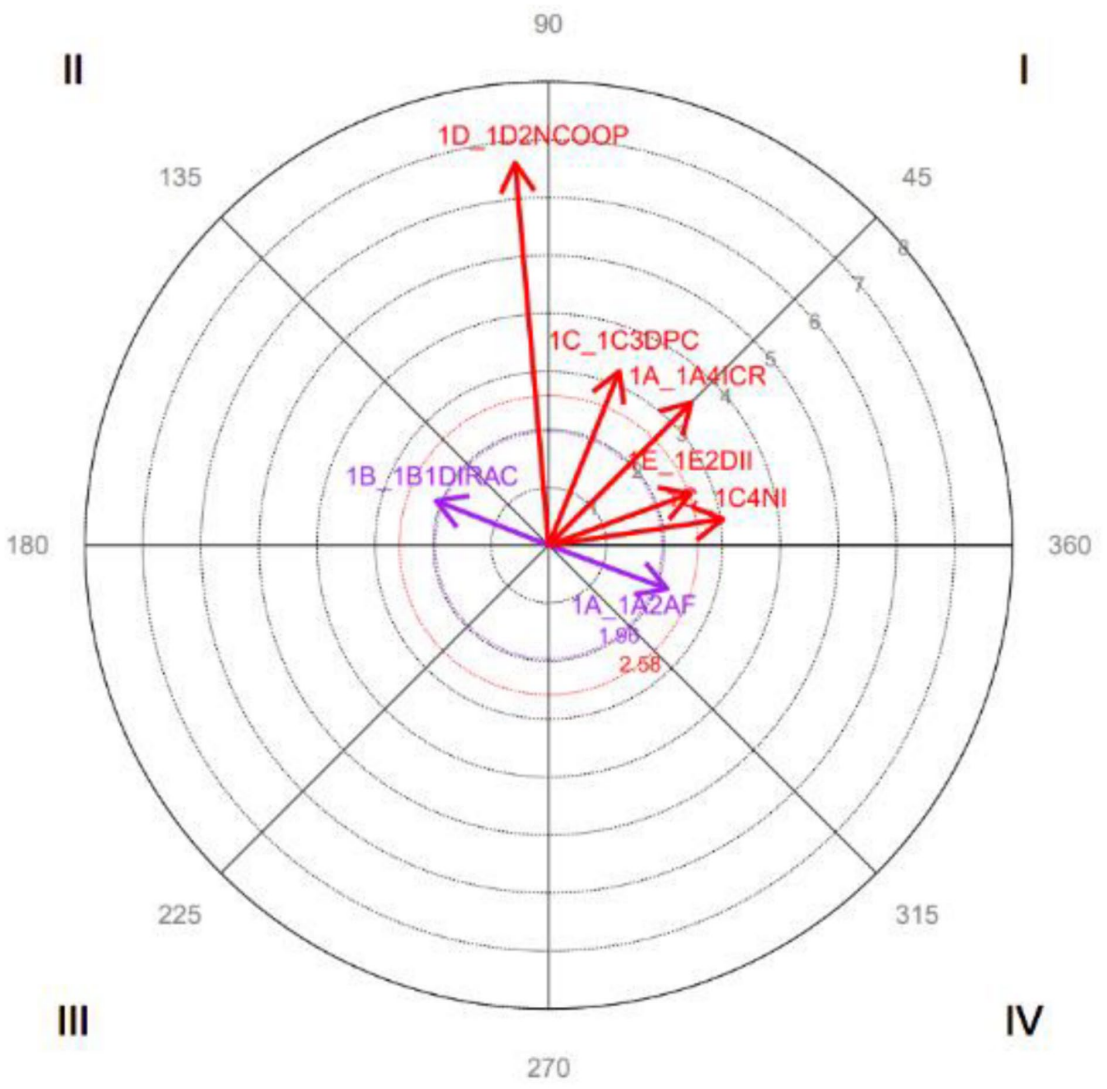
Graphical representation of the vectors corresponding to the 1A1AV category as focal and considering all categories as conditioned behaviors. Only significant vectors (length > 1.96) are included.

The results indicate the following relationships between focal and conditioned behavior (see Figure 2):

Quadrant I: *Verbal Aggression* is symmetrically activated with *Irrational Communication, Discourse of the Parties to a conflict, Incompatible Needs,* and D*ifferent interpretation of information*.
Quadrant II: *Verbal Aggression* inhibits *Perception of Effectiveness in Dealing with Conflict* and *Disinterest in the other Party in Conflict,* and these activate *Verbal Aggression.*
Quadrant IV: *Verbal Aggression* activates itself, although it is also inhibited by it at other times.

**Dimension 2: Focal behavior *Perception of the Conflictive Event***

Supplementary Table S2 presents the adjusted residuals of the lag sequential analysis, considering 2A1PEC (*Perception of the Conflicting Event*) as given behavior and all categories as conditioned behaviors, and considering prospective lags +1 to +5 and retrospective lags −1 to −5.

Supplementary Table S2 highlights the significantly adjusted residuals (>1.96, for a significance level of 0.05), from which the patterns of behavior are formed, and applies the conventional rules accordingly (Anguera et al., 2021). In all cases, the second rule has been applied.

The prospective patterns from given behavior 2A1PEC are:

**2A1PEC →** 1B3RP **→** 1B3RP **→**: *Perception of the Conflicting Event* activates *Power Relations*, which are maintained.
**2A1PEC →** Empty lag **→** 2A1PEC: *Perception of the Conflicting Event,* although not immediately, reinforces the *Perception of the Conflicting Event.*
**2A1PEC** ➔ Empty lag 2A2CPEN **→** 2A2CPEN **→**: *Perception of the Conflicting Event*, although not immediately, activates *Perceptible Behavior in the Natural Environment.*

The retrospective patterns before given behavior 2A1PEC are:

1A5CNR ← **2A1PEC**: *Perception of the Conflicting Event* is preceded by *Repetitive Negative Behaviors.*
1D1ICOP ← 1D1ICOP ←**2A1PEC**←: *Perception of the Conflicting Event* is preceded by *Imposing Beliefs on the Other Person,* which is maintained.
2A1PEC ← Empty lag ← **2A1PEC**: *Perception of the Conflicting Event,* although not immediately, is preceded by *Perception of the Conflicting Event.*
2A2CPEN ← **2A1PEC**: *Perception of the Conflicting Event* is preceded by *Perceptible Behavior in the Natural Environment.*
3C1UPCO ← 3C1UPCO ← **2A1PEC**: *Perception of the Conflicting Event* is preceded by *Assignment of One of the Parties.*

The parameters of the polar coordinate analysis have been calculated from the adjusted residuals, which are shown in Table 4.

**Table 4:** Values of the parameters corresponding to the analysis of polar coordinate, considering 2A1PEC as focal behavior, and all categories as conditioned behaviors.

Category	Quadrant	Prospective Zsum	Retrospective Zsum	Length	Signification	Angle
1A_1A5CNR	I	1.4	4.17	4.4	**	71.5
1B_1B3RP	I	2.64	2.18	3.42	**	39.57
1C_1C2NCIOP	I	3.66	1.48	3.95	**	22.02
1D_1D1ICOP	I	2.7	4.13	4.93	**	56.85
2A_2A1PEC	I	2.06	2.06	2.92	**	45
2A_2A2CPEN	I	2.98	2.78	4.08	**	43.04
2B_2B1FC	II	−1.84	2.09	2.79	**	131.37
2B_2B2FIG	IV	1.93	−1.51	2.45	*	321.88
3A_3A1PSFAPC	III	−1.13	−2.08	2.37	*	241.44
3D_3D1BIPGN	III	−1.47	−1.72	2.26	*	229.55
3E_3E1APBSI	III	−1.65	−1.53	2.25	*	222.8

Only conditioned behaviors in which significant and very significant values have been obtained are included.

Table 4, in addition to the prospective Zsum and retrospective Zsum values, shows the values corresponding to the length and angle of the vector in all cases where they are significant (length > 1.96, for a significance level of 0.05).

**Figure 3:**
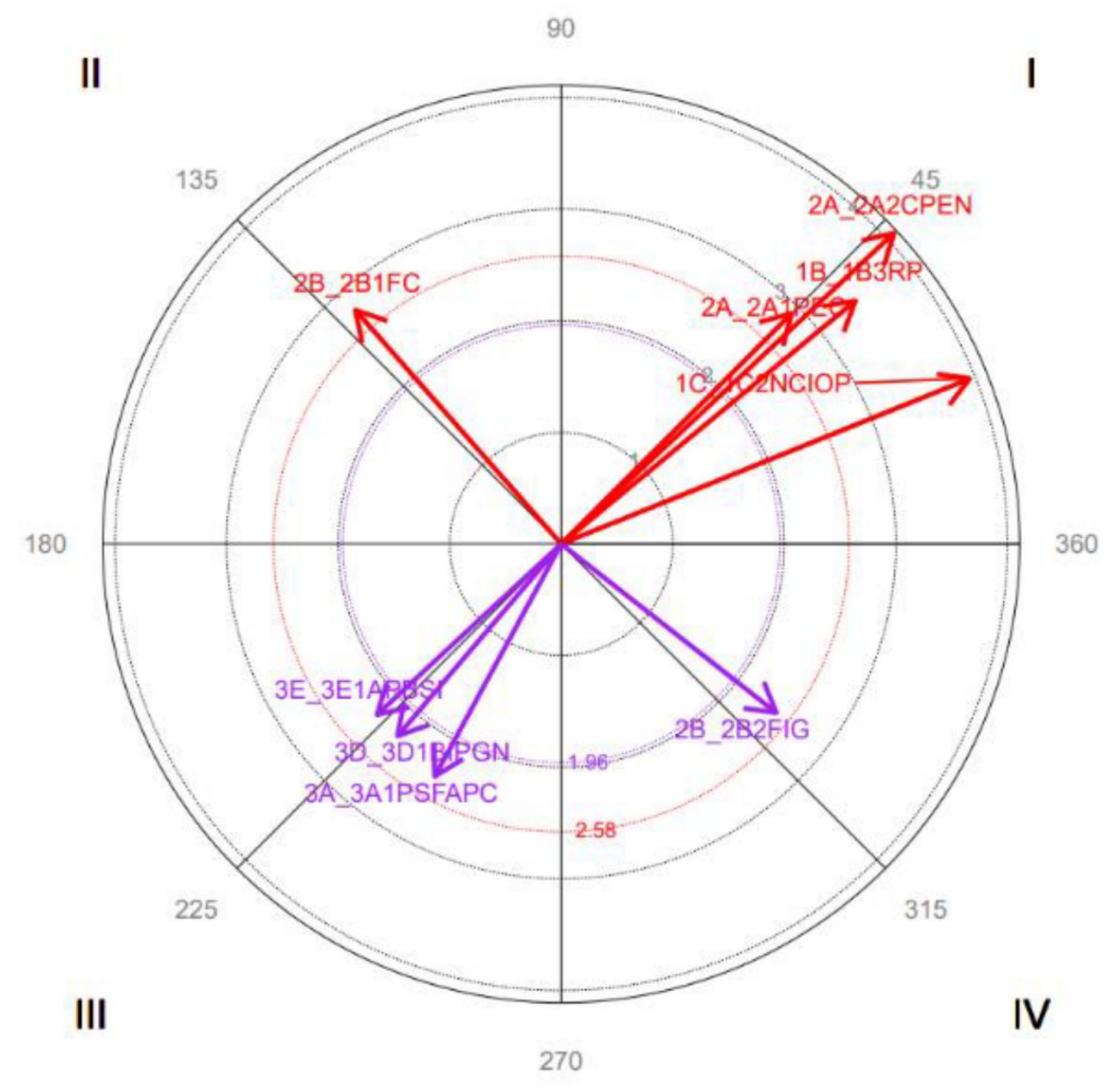
Graphical representation of the vectors corresponding to the 2A1PEC category as focal and considering all categories as conditioned behaviors. Only significant vectors (length > 1.96) are included.

The results indicate the following relationships between focal and conditioned behavior (see Figure 3):

Quadrant I: *Perception of the Conflicting Event* is symmetrically activated with *Repetitive Negative Behaviors, Power Relations, Disinterest in the other Party in the Conflict, Imposing Beliefs on the other Person, Perception of the Conflicting Event,* and *Perceptible Behavior in the Natural Environment.*
Quadrant II: *Perception of the Conflicting Event* inhibits *Lack of Trust,* and *Lack of Trust* activates it.
Quadrant III: *Perception of the Conflicting Event* is symmetrically inhibited with a *Mutually Friendly Solution, Low Intensity to Negotiate,* and *Intermediate Solution to the Conflict.*
Quadrant IV: *Perception of the Conflicting Event* activates *Lack of Identity with the Group*, although it is also inhibited by it at other times.

**Dimension 3: Focal behavior *Mutually Friendly Solution***

Supplementary Table S3 presents the adjusted residuals of the lag sequential analysis, considering 3A1PSFAPC (*Mutually Friendly Solutions*) as given behavior and all categories as conditioned behaviors, and considering prospective lags +1 to +5 and retrospective lags −1 to −5.

Supplementary Table S3 highlights the significantly adjusted residuals (>1.96, for a significance level of 0.05), from which the patterns of behavior are formed, and applying the conventional rules to this effect (Anguera et al., 2021). In all cases, the second has been applied.

The prospective patterns based on given behavior 3A1PSFAPC are:

**3A1PSFAPC →** 3A1PSFAPC: *Mutually Friendly Solution* activates itself.
**3A1PSFAPC →** Empty lag **→** 2C2TCA: *Mutually Friendly Solution, although* not immediately, activates *Associative Behavior Tendency.*
**3A1PSFAPC →** Empty lag **→** 3B1SIP **→** 3B1SIP **→**: *Mutually Friendly Solution*, but not immediately, activates *Satisfaction of Self-Interest*, which remains.
**3A1PSFAPC →** 4B3DPEAC: *Mutually Friendly Solution* activates *Perception of effectiveness in dealing with conflict.*
**3A1PSFAPC →** 4B4FCSC: *Mutually Friendly Solution* activates *Awareness of the Conflict Situation.*
**3A1PSFAPC →** Empty lag **→** 4B2TPC **→** 4B2TPC **→** 4B2TPC **→**: *Mutually Friendly Solution,* although not immediately, *Perspective-taking on the Conflict*, which is sustained for a time.

The retrospective patterns before given behavior 3A1PSFAPC are:

3A1PSFAPC ← **3A1PSFAPC:** *Mutually Friendly Solution* precedes itself.
3B1SIP ← **3A1PSFAPC:** *Mutually Friendly Solution* is preceded by *Satisfaction of Self-Interest.*
4B2TPC ← **3A1PSFAPC:** *Mutually Friendly Solution* is preceded by *Perspective-taking on the Conflict.*
1C1CO ← Empty lag ← **3A1PSFAPC:** *Mutually Friendly Solution*, although not immediately, is preceded by *Contrasting objectives.*
4A1EPC ← 4A1EPC ← **3A1PSFAPC:** *Mutually Friendly Solution* is preceded by *Empathy with the other Party in Conflict.*
4D1EA ← Empty lag ← **3A1PSFAPC:** *Mutually Friendly Solution*, although not immediately, is preceded by *Active Listening*.

The parameters of the polar coordinate analysis have been calculated from the adjusted residuals, which are shown in Table 5.

**Table 5:** Values of the parameters corresponding to the polar coordinate analysis, considering 3A1PSFAPC as focal behavior, and all categories as conditioned behaviors.

Category	Quadrant	Prospective Zsum	Retrospective Zsum	Length	Signification	Angle
1B_1B1DIRAC	III	−2.52	−1.92	3.17	**	217.25
1B_1B2FAI	III	−0.31	−1.97	2	*	260.94
1B_1B3RP	III	−1.4	−1.4	1.97	*	225
1C_1C4NI	II	−1.4	1.75	2.24	*	128.58
2A_2A1PEC	III	−2.08	−1.13	2.37	*	208.56
2B_2B2FIG	III	−1.4	−1.4	1.97	*	225
3A_3A1PSFAPC	I	4.56	4.56	6.45	**	45
3B_3B1SIP	I	2.81	1.52	3.19	**	28.43
3D_3D1BIPGN	IV	1.44	−1.46	2.05	*	314.6
3E_3E1APBSI	I	3.38	3.97	5.21	**	49.54
4A_4A1EPC	I	1.74	5.58	5.84	**	72.68
4A_4A2CPS	I	2.81	1.71	3.29	**	31.37
4B_4B2TPC	I	5.14	1.35	5.31	**	14.7
4B_4B3DPEAC	IV	2.33	−0.98	2.53	*	337.25
4B_4B4FCSC	I	4.61	4.61	6.53	**	45
4D_4D2UTVA	II	−0.8	1.89	2.05	*	112.87

Only conditioned behaviors in which significant and very significant values have been obtained are included.

Table 5, in addition to the prospective Zsum and retrospective Zsum values, shows the values corresponding to the length and angle of the vector in all cases where they are significant (length > 1.96, for a significance level of 0.05).

**Figure 4:**
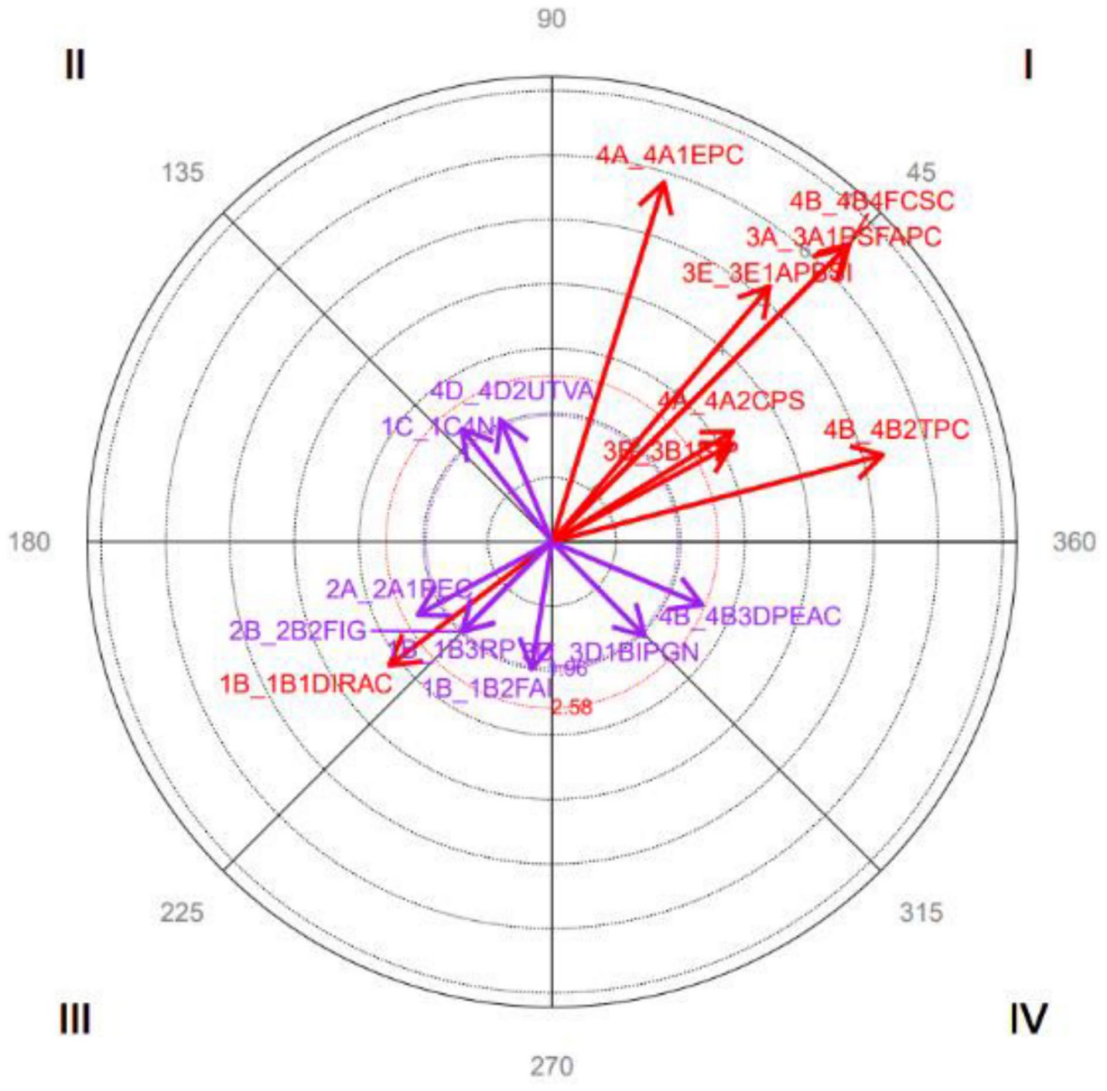
Graphical representation of the vectors corresponding to the 3A1PSFAPC category as focal and considering all categories as conditioned behaviors. Only significant vectors (length > 1.96) are included.

The results indicate the following relationships between focal and conditioned behavior (see Figure 4):

Quadrant I: *Mutually Friendly Solution* is activated symmetrically with *Mutually Friendly Solution, Satisfaction of Self-interest, Empathy with the other Party in Conflict, Prosocial Behavior, Perspective-taking on the Conflict,* and *Awareness of the Conflict Situation.*
Quadrant II: *Mutually Friendly Solution* inhibits *Incompatible Needs* and *Proper Tone of Voice*, and these activate it.
Quadrant III: *Mutually Friendly Solution* symmetrically inhibits *Insufficient Coping Resources Inter-Institutional Disarticulation, Power Relations, Perception of the Conflicting Event,* and *Lack of Identity with the Group*.
Quadrant IV: *Mutually Friendly Solution* activates Low *Intensity to Negotiate* and *Insufficient Coping Resources,* and these inhibit it.

**Dimension 4: Focal behavior *Empathy with the Other Party in Conflict***

Supplementary Table S4 presents the adjusted residuals of the sequential analysis of lags, considering 4A1EPC (*Empathy with the Other Party in Conflict*) as given behavior and all categories as conditioned behaviors, and considering prospective lags +1 to +5 and retrospective lags −1 to −5.

Supplementary Table S4 highlights the significantly adjusted residuals (>1.96, for a significance level of 0.05), from which the patterns of behavior are formed, and applies the conventional rules accordingly (Anguera et al., 2021). In all cases, the second rule has been applied.

The prospective patterns based on given 4A1EPC behavior are:

**4A1EPC →** 1C4NI: *Empathy with the other Party in Conflict* activates *Incompatible Needs.*
**4A1EPC →** 3A1PSFAPC **→** 3A1PSFAPC **→**: *Empathy with the Other Party in the Conflict* activ*ates Mutually Friendly Solution*, which is maintained.
**4A1EPC →** Empty lag **→** 4B1RE: *Empathy with the Other Party in the Conflict*, though not immediately, activates [*Auto] Regulates Emotions.*
**4A1EPC →** 4B4FCSC **→** 4B4FCSC **→**: *Empathy with the other Party in Conflict* activates *Awareness of the conflict situation*, which is maintained.
**4A1EPC →** 4C1APCC **→** 4C1APCC **→**: *Empathy with the other Party in Conflict* activates *Conflict Analysis/Critical Thinking*, which is maintained.

The retrospective patterns before given behavior 4A1EPC are:

1C4N1 ← **4A1EPC**: *Empathy with the other Party in Conflict* is preceded by *Incompatible Needs.*
4B2TPC ← Empty lag ← **4A1EPC**: *Empathy with the other Party in the Conflict*, though not immediately, is preceded by *Perspective-taking on the Conflict.*
4B4FCSC ← 4B4FCSC ← **4A1EPC**: *Empathy with the other Party in the Conflict* is preceded by *Awareness of the conflict situation*, which is maintained.
4D1EA ← **4A1EPC**: *Empathy with the other Party in the Conflict* is preceded by *Active Listening.*
4D2UTVA ← **4A1EPC**: *Empathy with the other Party in the Conflict* is preceded by a Proper *Tone of Voice.*

The parameters of the polar coordinate analysis have been calculated from the adjusted residuals, which are shown in Table 6.

**Table 6:** Parameter values corresponding to polar coordinate analysis, considering 4A1EPC as focal behavior, and all categories as conditioned behaviors.

Category	Quadrant	Prospective Zsum	Retrospective Zsum	Length	Signification	Angle
1B_1B1DIRAC	III	−1.94	−0.54	2.02	*	195.62
2C_2C2TCA	IV	2.76	−0.33	2.78	**	353.13
3A_3A1PSFAPC	I	5.58	1.74	5.84	**	17.32
3B_3B1SIP	I	2.15	0.03	2.15	*	0.76
3E_3E1APBSI	II	−1.13	4.61	4.75	**	103.8
4A_4A2CPS	I	3.19	2.3	3.94	**	35.83
4B_4B2TPC	I	2.75	2.77	3.9	**	45.17
4B_4B4FCSC	I	4.01	4.01	5.67	**	45
4C_4C1APCC	I	1.74	1.73	2.45	*	44.84
4D_4D1EA	II	−0.33	2.77	2.79	**	96.83
4D_4D2UTVA	II	−0.47	3.92	3.95	**	96.86

Only conditioned behaviors in which significant and very significant values have been obtained are included.

Table 6, in addition to the prospective Zsum and retrospective Zsum values, shows the values corresponding to the length and angle of the vector in all cases where they are significant (length > 1.96, for a significance level of 0.05).

**Figure 5:**
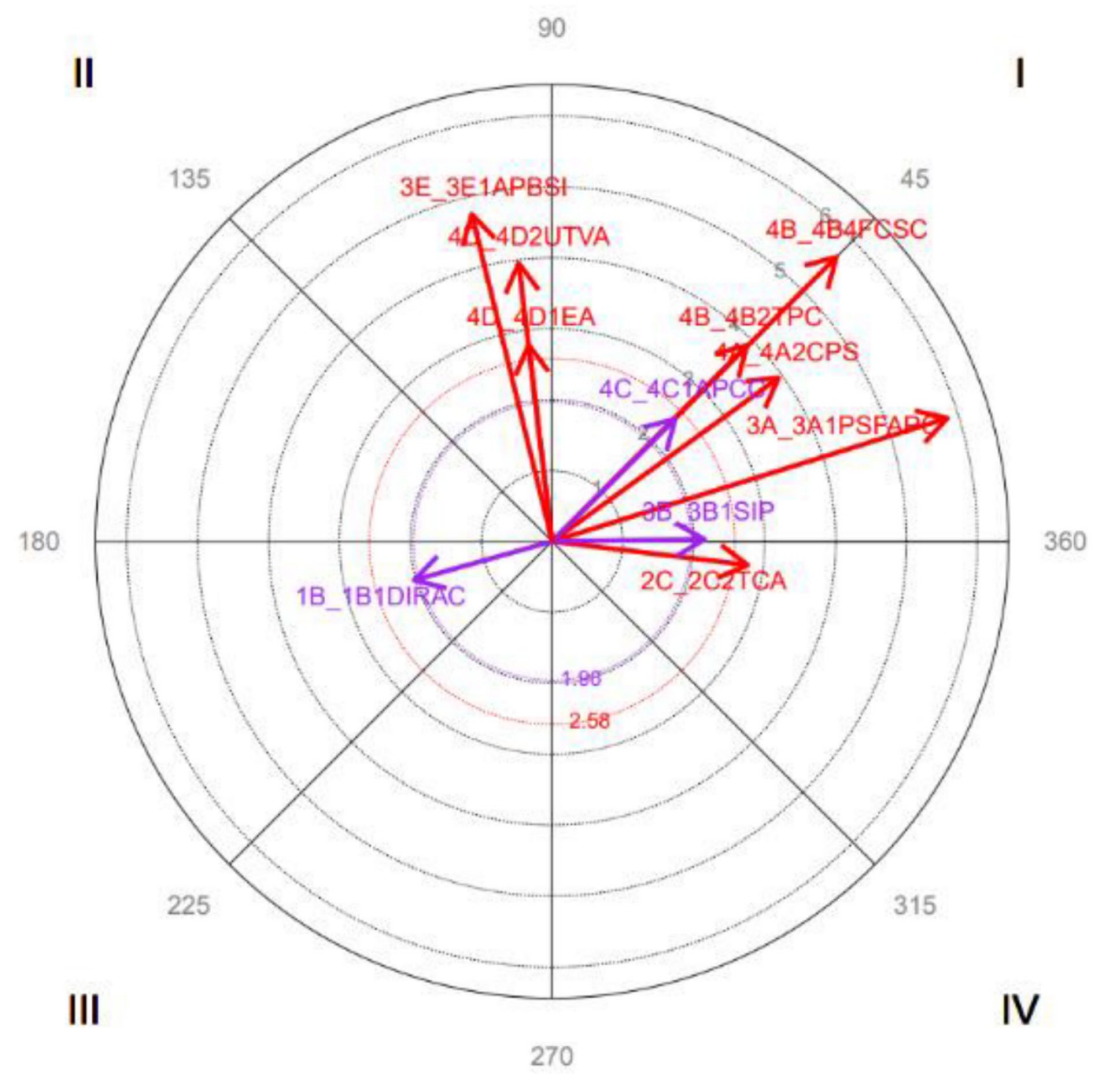
Graphical representation of the vectors corresponding to the 4A1EPC category as focal and considering all categories as conditioned behaviors. Only significant vectors (length > 1.96) are included.

The results indicate the following relationships between focal and conditioned behavior (see Figure 5):

Quadrant I: *Empathy with the other Party in the Conflict* is symmetrically activated with *Mutually Friendly Solutions, Satisfaction of Self-Interest, Prosocial Behavior, Perspective-taking on the Conflict, Awareness of the Conflict Situation,* and *Conflict Analysis/Critical Thinking*.
Quadrant II: *Empathy with the other Party in Conflict* inhibits *Intermediate Solution to the Conflict, Active Listening*, and *Proper Tone of Voice,* and these activate it.
Quadrant III: *Empathy with the other Party in Conflict* reciprocally inhibits I*nsufficient Coping Resources*.
Quadrant IV: *Empathy with the other Party in Conflict* activates an *Associative Behavior Tendency*, which in turn inhibits it.

## 4 Discussion

This study investigated behavioral patterns contributing to conflicts within Costa Rican secondary school classrooms and the strategies employed by teachers for conflict management. Two types of analysis—sequential delay analysis and polar coordinate analysis—were conducted, which helped in identifying 342 relationships between 38 criterion behaviors and their corresponding conditioned behaviors.

(1) The results show how *Verbal Aggression* (1A1AV) activates itself and prospectively generates both *Physical Assault* and *Irrational Communication*, and later triggers the emergence of the *Discourse of the Parties to a Conflict*, based on the *Different Interpretation of Information* they perform. The relationship between *Verbal Aggression* and *Physical Assault* as well as between *Verbal Aggression* and *Irrational Communication* is recurrently clear, both prospectively and retrospectively.

These relational findings contribute to the development of initiatives to strengthen the training processes of teachers in the Costa Rican educational system, as the communication skills they mainly use—according to their own narratives—are not sufficient resources for managing conflicts. Therefore, teachers need to improve both conceptual and practical training in conflict management and develop skills to properly manage the classroom climate, as they lack knowledge about the principles and use of conflict mediation that needs to be addressed (Alves and Pinto da Costa, 2021).

(2) In the results, it is demonstrated how the *Perception of the Conflicting Event* (2A1PEC) activates *Power Relations*, which intensify said *Perception of the Conflicting Event*. In turn, it is preceded by *Repetitive Negative Behaviors* and the Imposition of Beliefs, and *Lack of Trust* activates it. The *Perception of the Conflicting Event* activates *Lack of Identity with the Group*, and there is mutual inhibition with the *Mutually Friendly Solution* and *Low Intensity to Negotiate*.

These results, besides showing the negative perception of conflict in the classroom context and the resources teachers must manage them, demonstrate the need for teachers to differentiate the specific elements—such as perception—that influence the emergence of conflict, to focus the intervention strategy according to the dimension in which it arises.

This is one of the scopes offered by the Integrated Circular Model of Conflict (Bonilla et al., 2020), which enables, through phase analysis and considering teachers’ narratives, the recognition and differentiation of individual, group, and social aspects that may be factors influencing the emergence of conflicts in the classroom. In relation to the theoretical framework, the interviews with teachers indicate that, by failing to make such differentiation, it results in a perception of classrooms as environments of complex relationships, which poses new challenges. This supports the role of perceptions in conflict development (Grasa, 1987).

(3) The results indicate that *Mutually Friendly Solutions* (3A1PSFAPC) activate themselves and subsequently generate *Associative Behavior Tendency*, while activating *Satisfaction of Self-Interest*, *Empathy with the Other Party in Conflict, Prosocial Behavior*, and *Perspective-Taking on the Conflict*. In turn, they are arrived at after *Perception in Dealing with Conflict*, *Perspective-Taking on the Conflict*, and *Awareness of the Conflict Situation*. Finally, *Mutually Friendly Solutions* inhibit *Insufficient Coping Resources*, *Inter-Institutional Disarticulation, Power Relations, Perception of the Conflicting Event*, and *Lack of Identity with the Group*, and these factors reciprocally inhibit them.

Another contribution that the research makes to the field of conflict management in Costa Rican education is to show that the *Mutually Friendly Solution* behavior represents one of the most effective management strategies according to teachers, confirming that it is within the desirable strategies in mediation processes, according to Montes et al. (2014).

The study’s findings regarding criterion behavior activation align with Montes et al. (2014) regarding the commitment strategy, which seeks to reduce perceived differences, maximize gains for all parties, and facilitate swift conflict resolution.

Therefore, training teachers in coping strategies—such as *Mutually Friendly Solution*—also allows for the enhancement of other skills such as associative work, *Awareness of the Conflict Situation*, and perception of one’s own resources to manage conflicts, elements that were evidenced as lacking in the previous section.

(4) The results regarding the focal behavior *Empathy with the Other Party in Conflict* (4AIEPC) reveal that it first activates *Incompatible Needs, Awareness of the Conflict Situation*, and Conflict *Analysis/Critical Thinking*, which may be followed by *[Auto] Regulates Emotions*. It reciprocally activates with *Mutually Friendly Solution*, *Satisfaction of Self-Interest, Prosocial Behavior, Perspective-Taking on the Conflict, Awareness of the Conflict Solution*, and *Conflict Analysis/Critical Thinking*. Before its occurrence, the categories *Incompatible Needs* and *Active Listening* are detected. Likewise, it reciprocally inhibits with *Insufficient Coping Resources*.

In this regard, although teachers indicated that *Empathy with the Other Party in Conflict* is one of the strategies they use the most, they also pointed out that they do so “intuitively,” without prior knowledge of coping strategies. This confirms the findings of Calderón et al. (2014), regarding teachers’ responses to conflict situations that arise in the classroom, which vary according to the specificity of the conflict and the emotional moment but not as a planned or specific strategy.

The results, therefore, can guide actions and policy initiatives for structuring teacher training processes regarding conflict management in the classroom, as they have evidenced both the training gaps and the strategies required to improve teaching-learning dynamics, starting from the pursuit of good coexistence environments.

While this research is methodologically robust, drawing conclusions about how coping strategies, conflict mediation, and emotional regulation influence teachers’ perception of conflict requires further investigation. Limitations include the focus group’s size, the educational system’s historical context, and the Costa Rican Ministry of Public Education’s constraints on teacher training participation.

## 5 Conclusion

This study investigates behavioral relationships influencing the development, emergence, and management of conflicts within the classroom setting. Employing a novel mixed-methods approach for focus group analysis, the research examines both prospective and retrospective behavioral activation relationships. Key findings include:

- *Verbal Aggression* serves as a catalyst for conflict escalation, hindering effective resolution strategies.
- Individual perception significantly influences conflict emergence and subsequent management by educators. Teachers frequently report a lack of skills and strategies for intervention, leading to behavioral inaction in response to negative student behavior. This inaction risks perpetuating or intensifying conflict dynamics.
- Conversely, the pursuit of solutions benefiting all involved parties positively activates teachers’ perceptions of their conflict management efficacy. This leads to heightened *Awareness of the Conflict Situation* and improved perspective-taking.
- Empathy and *Active Listening* exhibit retrospective activation, facilitating critical conflict analysis. Furthermore, these techniques promote emotional regulation through modulated tone of voice, thus enhancing conflict management outcomes.

## 6 Future lines of research

Future studies could investigate the factors contributing to classroom conflicts, as highlighted in this research. First, additional classroom observations may reveal context-specific behavioral dynamics influencing conflict emergence. Second, research could determine the combined efficacy of coping strategies, conflict mediation, and emotional regulation in enhancing a teacher’s conflict resolution abilities. Third, broader teacher participation in studies might illuminate potential deficiencies within current training programs. Informed by these findings, researchers could recommend targeted improvements to teacher development in conflict analysis and management. Finally, expanding the scope of research to encompass individual, group, and social contributors to classroom conflict would facilitate the creation of multi-level conflict management strategies.

## Data Availability

Any anonymized data not published within the article will be shared by request from any qualified investigator.

## Footnotes

^1^https://www.mangold-international.com/en/products/software/gseq.html

## Supplementary material

SUPPLEMENTARY TABLE S1 Adjusted residuals corresponding to the lag sequential analysis considering 1A1AV as given behavior, all categories as conditioned behaviors, prospective lags R + 1 to R + 5, and retrospective lags R-1 to R-5.

SUPPLEMENTARY TABLE S2 Adjusted residuals corresponding to the lag sequential analysis considering 2A1PEC as given behavior, all categories as conditioned behaviors, prospective lags R + 1 to R + 5, and retrospective lags R-1 to R-5.

SUPPLEMENTARY TABLE S3 Adjusted residuals corresponding to the lag sequential analysis considering 3A1PSFAPC as given behavior, all categories as conditioned behaviors, prospective lags R + 1 to R + 5, and retrospective lags R-1 to R-5.

SUPPLEMENTARY TABLE S4 Adjusted residuals corresponding to the lag sequential analysis considering 4A1EPC as given behavior, all categories as conditioned behaviors, prospective lags R + 1 to R + 5, and retrospective lags R-1 to R-5.
