# Supplementary material for: Focus group on conflict management in the classroom in Secondary Education in Costa Rica: mixed methods approach
Source: Front Psychol. 2024 Oct 3;15:1407433. doi: 10.3389/fpsyg.2024.1407433 (PMC11483860; doi:10.3389/fpsyg.2024.1407433)
Supplement: SUPPLEMENTARY TABLE S1 — Adjusted residuals corresponding to the lag sequential analysis considering 1A1AV as given behavior, all categories as conditioned behaviors, prospective lags R + 1 to R + 5, and retrospective lags R-1 to R-5. [file Table_1.pdf]

Table 1

Adjusted residuals corresponding to the lag sequential analysis considering 1A1AV as given behavior, all categories as conditioned behaviors, prospective lags R+1 to R+5, and retrospective lags R-1 to R-5.

Codes	Lag -5 1A_1A1AV	Lag -4 1A_1A1AV	Lag -3 1A_1A1AV	Lag -2 1A_1A1AV	Lag -1 1A_1A1AV	Lag +1 1A_1A1AV	Lag +2 1A_1A1AV	Lag +3 1A_1A1AV	Lag +4 1A_1A1AV	Lag +5 1A_1A1AV
1A_1A1AV	-0,301	-0,301	-0,3	-0,299	**3,204**	**3,204**	-0,299	-0,3	-0,301	-0,301
1A_1A2AF	-0,338	-0,337	-0,336	-0,335	-0,334	**2,807**	-0,335	-0,336	-0,337	2,771
1A_1A3I	-0,301	-0,301	**3,185**	-0,299	-0,298	-0,298	-0,299	-0,3	-0,301	-0,301
1A_1A4ICR	-0,301	-0,301	-0,3	3,195	**3,204**	**3,204**	**3,195**	-0,3	-0,301	-0,301
1A_1A5CNR	-0,338	-0,337	-0,336	-0,335	-0,334	-0,298	-0,299	-0,259	-0,26	-0,26
1B_1B1DIRAC	2,149	0,637	-0,815	-0,831	0,574	-0,863	-0,866	-0,869	-0,872	-0,875
1B_1B2FAI	-0,51	-0,508	-0,531	-0,529	-0,528	-0,528	-0,529	-0,531	1,52	**3,561**
1B_1B3RP	**2,475**	-0,37	-0,369	-0,368	**2,508**	-0,367	-0,368	-0,369	-0,37	-0,371
1C_1C1CO	-0,26	-0,26	-0,259	-0,258	-0,257	-0,257	-0,258	**3,754**	-0,26	-0,212
1C_1C2NCIOP	-0,431	-0,43	**2,063**	2,07	-0,426	-0,426	-0,427	-0,429	-0,43	-0,431
1C_1C3DPC	-0,26	**3,743**	**3,754**	-0,258	-0,257	-0,257	**3,765**	-0,259	-0,26	-0,26
1C_1C4NI	-0,371	**2,483**	-0,369	-0,368	-0,367	-0,367	-0,368	**2,492**	**5,337**	-0,371
1D_1D1ICOP	-0,212	-0,211	-0,211	-0,21	-0,21	-0,21	-0,21	-0,211	-0,211	-0,212
1D_1D2NCOOP	**3,731**	**3,743**	**3,754**	**3,765**	-0,257	-0,257	-0,258	-0,259	-0,26	-0,26
1E_1E1EITD	-0,26	-0,26	-0,259	-0,258	-0,257	-0,257	**3,765**	-0,259	-0,26	-0,26
1E_1E2DII	-0,301	-0,301	-0,3	**3,195**	-0,298	-0,298	**3,195**	**3,185**	-0,301	-0,301
2A_2A1PEC	1,326	-0,579	1,339	1,345	-0,573	-0,573	-0,553	-0,554	-0,533	-0,534
2A_2A2CPEN	1,415	-0,556	-0,554	-0,553	-0,551	1,441	-0,553	-0,554	-0,556	1,415
2B_2B1FC	-0,431	-0,43	-0,429	-0,427	-0,426	-0,426	-0,427	-0,429	-0,43	**2,048**
2B_2B2FIG	-0,371	-0,37	-0,369	-0,368	**2,508**	-0,367	-0,368	-0,369	-0,37	-0,371
2C_2C1FHS	-0,745	-0,743	-0,741	0,831	0,836	0,836	0,831	-0,741	-0,743	-0,745
2C_2C2TCA	-0,149	-0,149	-0,149	-0,148	-0,148	-0,148	-0,148	-0,149	-0,149	-0,149
2C_2C3TCD	-0,301	-0,301	-0,3	-0,299	-0,298	-0,298	-0,299	**3,185**	-0,301	-0,301
3A_3A1PSFAPC	-0,51	1,629	-0,507	-0,505	-0,504	-0,504	-0,505	-0,507	-0,508	-0,51
3B_3B1SIP	-0,459	-0,457	-0,456	-0,455	-0,453	-0,453	-0,455	-0,456	-0,457	1,885
3C_3C1UPCO	-0,338	-0,337	-0,336	-0,335	-0,334	-0,334	-0,335	-0,336	-0,337	2,771
3D_3D1BIPGN	-0,645	1,107	1,113	-0,639	-0,637	-0,637	-0,639	-0,641	-0,643	1,101
3E_3E1APBSI	-0,51	-0,508	-0,507	-0,505	-0,504	-0,504	-0,505	-0,507	1,629	-0,51
4A_4A1EPC	-0,301	-0,301	-0,3	-0,299	-0,298	-0,298	-0,299	-0,3	-0,301	-0,301
4A_4A2CPS	-0,558	1,422	-0,554	-0,553	-0,551	-0,551	-0,553	-0,554	-0,556	-0,558
4B_4B1RE	-0,301	-0,301	-0,3	-0,299	-0,298	-0,298	-0,299	-0,3	-0,301	-0,301
4B_4B2TPC	-0,149	-0,149	-0,149	-0,148	-0,148	-0,148	-0,148	-0,149	-0,149	-0,149
4B_4B3DPEAC	-0,26	-0,26	-0,259	-0,258	-0,257	-0,257	-0,258	-0,259	-0,26	-0,26
4B_4B4FCSC	-0,301	-0,301	-0,3	-0,299	-0,298	-0,298	-0,299	-0,3	-0,301	-0,301
4C_4C1APCC	-0,371	-0,37	-0,369	-0,368	-0,367	-0,367	-0,368	-0,369	**2,483**	-0,371
4D_4D1EA	-0,149	-0,149	-0,149	-0,148	-0,148	-0,148	-0,148	-0,149	-0,149	-0,149
4D_4D2UTVA	-0,212	-0,211	-0,211	-0,21	-0,21	-0,21	-0,21	-0,211	-0,211	-0,212
4D_4D3CI	-0,212	-0,211	-0,211	-0,21	-0,21	-0,21	-0,21	-0,211	-0,211	-0,212
