# Supplementary material for: Focus group on conflict management in the classroom in Secondary Education in Costa Rica: mixed methods approach
Source: Front Psychol. 2024 Oct 3;15:1407433. doi: 10.3389/fpsyg.2024.1407433 (PMC11483860; doi:10.3389/fpsyg.2024.1407433)
Supplement: SUPPLEMENTARY TABLE S2 — Adjusted residuals corresponding to the lag sequential analysis considering 2A1PEC as given behavior, all categories as conditioned behaviors, prospective lags R + 1 to R + 5, and retrospective lags R-1 to R-5. [file Table_2.pdf]

Table 2

Adjusted residuals corresponding to the lag sequential analysis considering 2A1PEC as given behavior, all categories as conditioned behaviors, prospective lags R+1 to R+5, and retrospective lags R-1 to R-5.

Codes	Lag -5 2A_2A1PEC	Lag -4 2A_2A1PEC	Lag -3 2A_2A1PEC	Lag -2 2A_2A1PEC	Lag -1 2A_2A1PEC	Lag +1 2A_2A1PEC	Lag +2 2A_2A1PEC	Lag +3 2A_2A1PEC	Lag +4 2A_2A1PEC	Lag +5 2A_2A1PEC
1A_1A1AV	-0,534	-0,533	-0,554	-0,553	-0,573	-0,573	1,345	1,339	-0,579	1,326
1A_1A2AF	1,238	-0,597	-0,621	-0,62	-0,643	1,084	-0,645	1,072	-0,649	1,06
1A_1A3I	-0,534	-0,533	-0,554	-0,553	1,352	-0,573	-0,575	-0,577	1,333	-0,58
1A_1A4ICR	-0,534	-0,533	-0,554	-0,553	-0,573	-0,573	-0,575	-0,577	1,333	-0,58
1A_1A5CNR	**3,074**	-0,597	1,157	1,163	**4,537**	3,277	1,345	-0,498	-0,5	-0,501
1B_1B1DIRAC	-1,421	-0,59	-0,678	0,101	0,714	-1,661	0,636	-0,906	0,616	-0,921
1B_1B2FAI	0,356	-0,901	0,189	-0,979	0,121	0,121	-1,019	-1,022	-1,025	-1,028
1B_1B3RP	-0,658	**2,714**	**2,573**	0,951	-0,706	**2,455**	**2,445**	-0,71	0,857	0,851
1C_1C1CO	-0,461	-0,46	-0,479	-0,477	1,722	1,722	-0,497	1,708	-0,5	-0,408
1C_1C2NCIOP	0,7	**2,174**	0,625	0,631	-0,82	0,556	0,551	0,545	**3,273**	**3,26**
1C_1C3DPC	-0,461	-0,46	-0,479	-0,477	1,722	1,722	1,715	1,708	-0,5	-0,501
1C_1C4NI	-0,658	-0,656	-0,683	-0,681	-0,706	-0,706	-0,708	0,863	-0,713	0,851
1D_1D1ICOP	**2,504**	-0,374	**5,187**	-0,389	**2,305**	-0,403	-0,404	**2,289**	-0,407	**4,955**
1D_1D2NCOOP	-0,461	-0,46	-0,479	-0,477	-0,495	1,722	-0,497	1,708	-0,5	-0,501
1E_1E1EITD	-0,461	1,904	-0,479	-0,477	-0,495	-0,495	-0,497	-0,498	1,701	1,694
1E_1E2DII	-0,534	-0,533	-0,554	-0,553	-0,573	-0,573	-0,575	-0,577	-0,579	1,326
2A_2A1PEC	0,098	1,232	1,113	**2,214**	-0,045	-0,045	**2,214**	1,113	1,232	0,098
2A_2A2CPEN	0,177	1,351	1,231	1,239	**2,225**	0,035	**3,307**	**2,204**	1,105	0,012
2B_2B1FC	**2,164**	0,706	0,625	0,631	0,556	-0,82	-0,822	-0,825	-0,827	-0,83
2B_2B2FIG	-0,658	-0,656	-0,683	-0,681	-0,706	0,874	0,868	0,863	0,857	0,851
2C_2C1FHS	1,44	-0,394	0,412	1,312	-1,416	1,177	-1,42	-1,425	1,147	-1,435
2C_2C2TCA	-0,265	-0,264	-0,275	-0,274	-0,284	-0,284	-0,285	-0,286	-0,287	-0,288
2C_2C3TCD	1,513	1,52	-0,554	-0,553	1,352	-0,573	1,345	-0,577	-0,579	1,326
3A_3A1PSFAPC	-0,903	-0,901	-0,937	-0,934	-0,97	-0,97	-0,973	0,203	-0,979	0,191
3B_3B1SIP	-0,813	0,578	0,497	-0,84	0,429	-0,872	-0,875	0,418	-0,88	0,406
3C_3C1UPCO	-0,599	-0,597	**2,935**	-0,62	**2,81**	1,084	-0,645	-0,647	-0,649	-0,651
3D_3D1BIPGN	-1,143	-0,106	-1,186	-0,182	-1,226	-1,226	0,703	-1,234	-1,238	-0,282
3E_3E1APBSI	-0,903	-0,901	-0,937	0,288	-0,97	0,214	-0,973	-0,976	-0,979	-0,982
4A_4A1EPC	-0,534	-0,533	-0,554	-0,553	-0,573	-0,573	-0,575	-0,577	-0,579	-0,58
4A_4A2CPS	0,177	0,183	-1,025	-1,022	0,035	0,035	-1,063	1,113	-1,07	0,012
4B_4B1RE	-0,534	-0,533	-0,554	-0,553	-0,573	-0,573	-0,575	-0,577	-0,579	-0,58
4B_4B2TPC	-0,265	-0,264	-0,275	-0,274	-0,284	-0,284	-0,285	-0,286	-0,287	-0,288
4B_4B3DPEAC	-0,461	-0,46	-0,479	-0,477	-0,495	-0,495	-0,497	-0,498	-0,5	-0,501
4B_4B4FCSC	-0,534	-0,533	-0,554	-0,553	-0,573	-0,573	-0,575	-0,577	-0,579	-0,58
4C_4C1APCC	-0,658	-0,656	0,945	0,951	-0,706	0,874	-0,708	-0,71	-0,713	-0,715
4D_4D1EA	-0,265	-0,264	-0,275	-0,274	-0,284	-0,284	-0,285	-0,286	-0,287	-0,288
4D_4D2UTVA	-0,376	-0,374	-0,39	-0,389	-0,403	-0,403	-0,404	-0,406	-0,407	-0,408
4D_4D3CI	-0,376	-0,374	-0,39	-0,389	-0,403	-0,403	-0,404	-0,406	-0,407	-0,408
PARTICIPANTE_P1	0,265	0,264	0,275	0,274	0,284	0,284	0,285	0,286	0,287	0,288
