# Supplementary material for: Focus group on conflict management in the classroom in Secondary Education in Costa Rica: mixed methods approach
Source: Front Psychol. 2024 Oct 3;15:1407433. doi: 10.3389/fpsyg.2024.1407433 (PMC11483860; doi:10.3389/fpsyg.2024.1407433)
Supplement: SUPPLEMENTARY TABLE S3 — Adjusted residuals corresponding to the lag sequential analysis considering 3A1PSFAPC as given behavior, all categories as conditioned behaviors, prospective lags R + 1 to R + 5, and retrospective lags R-1 to R-5. [file Table_3.pdf]

Table 3

Adjusted residuals corresponding to the lag sequential analysis considering 3A1PSFAPC as given behavior, all categories as conditioned behaviors, prospective lags R+1 to R+5, and retrospective lags R-1 to R-5.

Codes	Lag -5<br>3A_3A1PSFAPC	Lag -4<br>3A_3A1PSFAPC	Lag -3<br>3A_3A1PSFAPC	Lag -2<br>3A_3A1PSFAPC	Lag -1<br>3A_3A1PSFAPC	Lag +1<br>3A_3A1PSFAPC	Lag +2<br>3A_3A1PSFAPC	Lag +3<br>3A_3A1PSFAPC	Lag +4<br>3A_3A1PSFAPC	Lag +5<br>3A_3A1PSFAPC
1A_1A1AV	-0,51	-0,508	-0,507	-0,505	-0,504	-0,504	-0,505	-0,507	1,629	-0,51
1A_1A2AF	-0,572	-0,57	-0,568	-0,567	-0,565	-0,565	-0,567	-0,568	-0,57	1,341
1A_1A3I	-0,51	-0,508	-0,507	-0,505	-0,504	-0,504	-0,505	-0,507	-0,508	-0,51
1A_1A4ICR	-0,51	-0,508	1,636	1,643	-0,504	1,649	-0,505	-0,507	-0,508	-0,51
1A_1A5CNR	-0,572	1,347	-0,568	-0,567	-0,565	-0,504	-0,505	-0,438	-0,439	-0,44
1B_1B1DIRAC	-1,356	-0,488	-0,482	-1,404	-0,557	-1,46	-1,465	-0,613	-1,474	-0,626
1B_1B2FAI	-0,862	-0,86	-0,898	-0,895	-0,893	-0,893	-0,895	0,367	0,362	0,356
1B_1B3RP	-0,628	-0,626	-0,624	-0,622	-0,621	-0,621	-0,622	-0,624	-0,626	-0,628
1C_1C1CO	-0,44	-0,439	-0,438	**2,037**	-0,435	-0,435	-0,437	-0,438	-0,439	-0,359
1C_1C2NCIOP	-0,729	-0,727	-0,725	-0,723	-0,721	0,819	-0,723	-0,725	-0,727	-0,729
1C_1C3DPC	-0,44	-0,439	-0,438	-0,437	-0,435	-0,435	-0,437	-0,438	-0,439	-0,44
1C_1C4NI	1,123	1,129	1,135	-0,622	1,147	-0,621	-0,622	-0,624	-0,626	-0,628
1D_1D1ICOP	-0,359	-0,358	-0,356	-0,355	-0,354	-0,354	-0,355	-0,356	-0,358	-0,359
1D_1D2NCOOP	-0,44	-0,439	-0,438	-0,437	-0,435	-0,435	-0,437	-0,438	-0,439	-0,44
1E_1E1EITD	-0,44	-0,439	-0,438	-0,437	-0,435	-0,435	-0,437	-0,438	-0,439	-0,44
1E_1E2DII	-0,51	-0,508	-0,507	-0,505	-0,504	-0,504	1,643	-0,507	-0,508	-0,51
2A_2A1PEC	0,191	-0,979	0,203	-0,973	-0,97	-0,97	-0,934	-0,937	-0,901	-0,903
2A_2A2CPEN	0,27	0,276	-0,937	-0,934	-0,932	-0,932	-0,934	0,282	0,276	-0,943
2B_2B1FC	0,796	0,801	0,807	-0,723	-0,721	-0,721	-0,723	-0,725	0,801	0,796
2B_2B2FIG	-0,628	-0,626	-0,624	-0,622	-0,621	-0,621	-0,622	-0,624	-0,626	-0,628
2C_2C1FHS	-1,26	-0,296	**2,598**	1,646	-0,278	1,656	0,681	-0,29	-1,256	-1,26
2C_2C2TCA	-0,253	-0,252	-0,251	-0,251	-0,25	-0,25	**4,011**	-0,251	-0,252	-0,253
2C_2C3TCD	-0,51	-0,508	-0,507	-0,505	-0,504	-0,504	-0,505	-0,507	-0,508	-0,51
3A_3A1PSFAPC	**3,072**	1,769	1,778	0,466	**3,12**	**3,12**	0,466	1,778	1,769	**3,072**
3B_3B1SIP	0,666	-0,773	0,678	0,683	**2,145**	-0,766	**2,135**	**2,126**	2,117	0,666
3C_3C1UPCO	-0,572	-0,57	-0,568	1,36	-0,565	1,366	1,36	1,354	-0,57	-0,572
3D_3D1BIPGN	-1,091	-0,012	-1,084	-1,081	0,006	1,089	1,081	1,073	1,065	-1,091
3E_3E1APBSI	**3,072**	**3,084**	1,778	1,787	-0,852	1,796	0,466	0,461	1,769	**3,072**
4A_4A1EPC	**3,755**	1,629	-0,507	**3,791**	**3,803**	1,649	-0,505	-0,507	1,629	1,622
4A_4A2CPS	**2,697**	0,276	1,501	-0,934	0,293	1,518	0,288	**2,72**	1,492	0,27
4B_4B1RE	-0,51	-0,508	-0,507	1,643	-0,504	-0,504	-0,505	1,636	-0,508	-0,51
4B_4B2TPC	-0,253	-0,252	-0,251	-0,251	**4,022**	-0,25	**4,011**	**3,999**	**3,988**	-0,253
4B_4B3DPEAC	-0,44	-0,439	-0,438	-0,437	-0,435	**2,044**	2,037	-0,438	-0,439	**2,015**
4B_4B4FCSC	1,622	**3,767**	1,636	1,643	1,649	**3,803**	-0,505	1,636	1,629	**3,755**
4C_4C1APCC	-0,628	1,129	1,135	1,141	-0,621	1,147	1,141	1,135	-0,626	-0,628
4D_4D1EA	-0,253	-0,252	-0,251	**4,011**	-0,25	-0,25	-0,251	-0,251	-0,252	-0,253
4D_4D2UTVA	**2,64**	**2,649**	-0,356	-0,355	-0,354	-0,354	-0,355	-0,356	-0,358	-0,359
4D_4D3CI	-0,359	-0,358	-0,356	-0,355	-0,354	-0,354	-0,355	-0,356	-0,358	2,64
