# Supplementary material for: Focus group on conflict management in the classroom in Secondary Education in Costa Rica: mixed methods approach
Source: Front Psychol. 2024 Oct 3;15:1407433. doi: 10.3389/fpsyg.2024.1407433 (PMC11483860; doi:10.3389/fpsyg.2024.1407433)
Supplement: SUPPLEMENTARY TABLE S4 — Adjusted residuals corresponding to the lag sequential analysis considering 4A1EPC as given behavior, all categories as conditioned behaviors, prospective lags R + 1 to R + 5, and retrospective lags R-1 to R-5. [file Table_4.pdf]

Table 4

Adjusted residuals corresponding to the lag sequential analysis considering 4A1EPC as given behavior, all categories as conditioned behaviors, prospective lags R+1 to R+5, and retrospective lags R-1 to R-5.

Codes	Lag -5 4A_4A1EPC	Lag -4 4A_4A1EPC	Lag -3 4A_4A1EPC	Lag -2 4A_4A1EPC	Lag -1 4A_4A1EPC	Lag +1 4A_4A1EPC	Lag +2 4A_4A1EPC	Lag +3 4A_4A1EPC	Lag +4 4A_4A1EPC	Lag +5 4A_4A1EPC
1A_1A1AV	-0,301	-0,301	-0,3	-0,299	-0,298	-0,298	-0,299	-0,3	-0,301	-0,301
1A_1A2AF	-0,338	-0,337	-0,336	-0,335	-0,334	-0,334	-0,335	-0,336	-0,337	-0,338
1A_1A3I	-0,301	-0,301	-0,3	-0,299	-0,298	-0,298	-0,299	-0,3	-0,301	-0,301
1A_1A4ICR	-0,301	-0,301	-0,3	-0,299	-0,298	-0,298	-0,299	**3,185**	-0,301	-0,301
1A_1A5CNR	-0,338	-0,337	-0,336	-0,335	-0,334	-0,298	-0,299	-0,259	-0,26	-0,26
1B_1B1DIRAC	-0,802	-0,818	0,643	0,608	-0,846	-0,863	-0,866	-0,869	-0,872	-0,875
1B_1B2FAI	-0,51	-0,508	-0,531	-0,529	-0,528	-0,528	-0,529	-0,531	-0,533	1,513
1B_1B3RP	-0,371	-0,37	-0,369	-0,368	-0,367	-0,367	-0,368	-0,369	-0,37	-0,371
1C_1C1CO	-0,26	-0,26	-0,259	-0,258	-0,257	-0,257	-0,258	-0,259	-0,26	-0,212
1C_1C2NCIOP	-0,431	-0,43	-0,429	-0,427	-0,426	-0,426	-0,427	-0,429	-0,43	-0,431
1C_1C3DPC	-0,26	-0,26	-0,259	-0,258	-0,257	-0,257	-0,258	-0,259	-0,26	-0,26
1C_1C4NI	-0,371	-0,37	-0,369	-0,368	**2,508**	**2,508**	-0,368	-0,369	-0,37	-0,371
1D_1D1ICOP	-0,212	-0,211	-0,211	-0,21	-0,21	-0,21	-0,21	-0,211	-0,211	-0,212
1D_1D2NCOOP	-0,26	-0,26	-0,259	-0,258	-0,257	-0,257	-0,258	-0,259	-0,26	-0,26
1E_1E1EITD	-0,26	-0,26	-0,259	-0,258	-0,257	-0,257	-0,258	-0,259	-0,26	-0,26
1E_1E2DII	-0,301	-0,301	-0,3	-0,299	-0,298	-0,298	-0,299	-0,3	-0,301	-0,301
2A_2A1PEC		-0,579	-0,577	-0,575	-0,573	-0,573	-0,553	-0,554	-0,533	-0,534
2A_2A2CPEN	-0,558	1,422	-0,554	-0,553	-0,551	-0,551	-0,553	-0,554	-0,556	-0,558
2B_2B1FC	-0,431	-0,43	**2,063**	-0,427	-0,426	-0,426	-0,427	-0,429	-0,43	-0,431
2B_2B2FIG	-0,371	-0,37	-0,369	-0,368	-0,367	-0,367	-0,368	-0,369	-0,37	-0,371
2C_2C1FHS	-0,745	0,819	-0,741	-0,738	0,836	-0,736	0,831	0,825	-0,743	0,813
2C_2C2TCA	-0,149	-0,149	-0,149	-0,148	-0,148	-0,148	-0,148	6,764	-0,149	-0,149
2C_2C3TCD	-0,301	-0,301	-0,3	-0,299	-0,298	-0,298	-0,299	-0,3	-0,301	-0,301
3A_3A1PSFAPC	1,622	1,629	-0,507	-0,505	1,649	**3,803**	**3,791**	-0,507	1,629	3,755
3B_3B1SIP	1,885	-0,457	-0,456	-0,455	-0,453	1,914	1,907	1,9	-0,457	-0,459
3C_3C1UPCO	-0,338	-0,337	**2,789**	-0,335	-0,334	-0,334	-0,335	**2,789**	-0,337	-0,338
3D_3D1BIPGN	-0,645	-0,643	-0,641	1,119	-0,637	-0,637	-0,639	-0,641	1,107	1,101
3E_3E1APBSI	1,622	**3,767**	1,636	1,643	1,649	-0,504	-0,505	-0,507	-0,508	-0,51
4A_4A1EPC	-0,301	-0,301	-0,3	-0,299	-0,298	-0,298	-0,299	-0,3	-0,301	-0,301
4A_4A2CPS	-0,558	**5,377**	-0,554	-0,553	1,441	1,441	1,435	-0,554	**3,399**	1,415
4B_4B1RE	-0,301	3,175	-0,3	-0,299	-0,298	-0,298	**3,195**	-0,3	-0,301	-0,301
4B_4B2TPC	-0,149	-0,149	-0,149	**6,782**	-0,148	-0,148	-0,148	-0,149	**6,745**	-0,149
4B_4B3DPEAC	-0,26	-0,26	-0,259	-0,258	-0,257	-0,257	-0,258	-0,259	-0,26	-0,26
4B_4B4FCSC	**3,165**	-0,301	-0,3	**3,195**	**3,204**	**3,204**	**3,195**	-0,3	-0,301	**3,165**
4C_4C1APCC	**2,475**	-0,37	**2,492**	-0,368	-0,367	-0,367	**2,5**	**2,492**	-0,37	-0,371
4D_4D1EA	-0,149	-0,149	-0,149	-0,148	**6,8**	-0,148	-0,148	-0,149	-0,149	-0,149
4D_4D2UTVA	-0,212	-0,211	4,69	**4,703**	-0,21	-0,21	-0,21	-0,211	-0,211	-0,212
4D_4D3CI	4,663	-0,211	-0,211	-0,21	-0,21	-0,21	-0,21	-0,211	-0,211	-0,212
