# Supplementary material for: Focus group on conflict management in the classroom in Secondary Education in Costa Rica: mixed methods approach
Source: Front Psychol. 2024 Oct 3;15:1407433. doi: 10.3389/fpsyg.2024.1407433 (PMC11483860; doi:10.3389/fpsyg.2024.1407433)
Supplement: Supplementary file 5 [file Data_Sheet_1.pdf]

Supplemenrary material
Question 1 Coded answers

Participant	Textual Units	1. Typology of the conflict					2. Aspects that affect the emergence of the conflict			3. Coping strategy					4. Teaching skills to manage conflict			
		A. Interpersonal	B. Structural	C. Interests	D. Value	E. Information	A. Individuals	B. Group	C. Social	A. Integration	B. Domination	C. Servility	D. Evitation	E. Commitment	A. Social	B. Emotional for conflict negotiation	C. Cognitive	D. Comunication
1	1	1A5CNR																
	2	1A5CNR																
	3						2A1PEC											
	4	1A5CNR																
	5																	
	6						2A1PEC											
2	7			1C1CO														
	8		1B1DIRAC															
	9			1C4NI						3A1PSFAPC					4A2CPS			
	10			1C2NCIOP														
3	11					1E2DII												
	12		1B1DIRAC															
	13	1A1AV																
	14	1A2AF																
	15			1C3DPC														
4	16			1C1CO														
	17			1C4NI														
5	18		1B2FAI					2B1FC				3C1UPCO						
	19							2B1FC										
	20					1E1EITD												
	21								2C1FHS									
6	22	1A5CNR					2A2CPEN											
	23			1C1CO								3C1UPCO						
	24																	
	25						2A1PEC											
7	26	1A5CNR																
	27			1C2NCIOP														
	28			1C2NCIOP														
	29			1C2NCIOP														
	30				1D1ICOP													
	31						2A1PEC						3D1BIPGN					
8	32		1B2FAI									3C1UPCO						
	33						2A1PEC											
9	34			1C3DPC											4A2CPS			
	35						2A1PEC											
	36	1A2AF																
	37		1B1DIRAC															
10	38				1D1ICOP						3B1SIP							
	39		1B1DIRAC															
	40		1B1DIRAC															
	41		1B1DIRAC				2A1PEC											
11	42																	
	43				1D2NCOOP													
	44			1C3DPC									3D1BIPGN					
	45				1D2NCOOP													
	46	1A4ICR							2C1FHS									
12	47	1A1AV																
	48	1A1AV																
	49	1A4ICR							2C1FHS									
	50					1E2DII												
	51																	
	52			1C4NI														
	53		1B2FAI															
13	54	1A2AF									3B1SIP							
	55	1A2AF									3B1SIP							
	56					1E2DII												
	57	1A2AF					2A2CPEN											
	58																	
	59							2B2FIG										
	60																	
	61	1A4ICR																
14	62		1B3RP					2B2FIG										
	63	1A3I																
	64		1B3RP															
	65				1D2NCOOP													
	66								2C1FHS									
	67	1A3I							2C1FHS									
