# Supplementary material for: Focus group on conflict management in the classroom in Secondary Education in Costa Rica: mixed methods approach
Source: Front Psychol. 2024 Oct 3;15:1407433. doi: 10.3389/fpsyg.2024.1407433 (PMC11483860; doi:10.3389/fpsyg.2024.1407433)
Supplement: Supplementary file 6 [file Data_Sheet_2.pdf]

Supplemenrary material
Question 2 Coded answers

		1. Tipology of the conflict					2. Aspects that affect the emergence of the conflict			3. Coping strategy					4. Teaching skills to manage conflict			
Participant	Textual Units	A. Interpersonal	B. Structural	C. Interests	D. Value	E. Information	A. Individual	B. Group	C. Social	A. Integration	B. Domination	C. Servilility	D. Evitation	E. Commitment	A. Social	B. Emotional for conflict negotiation	C. Cognitive	D. Comunication
1	1								2C1FHS									
	2																	
	3		1B3RP															
	4		1B1DIRAC									3C1UPCO						
	5								2C1FHS									
2	6								2C3TCD		3B1SIP							
	7						2A1PEC										4C1APCC	
	8													3E1APBSI			4C1APCC	
	9						2A2CPEN											
	10						2A1PEC											
3	11								2C1FHS									
	12												3D1BIPGN					
4	13							2B2FIG										
	14			1C2NCIOP				2B2FIG										
	15			1C2NCIOP														
5	16								2C1FHS							4B1RE		
	17								2C1FHS						4A2CPS			
	18								2C1FHS						4A2CPS			
	19								2C1FHS									
	20								2C1FHS									
6	21		1B1DIRAC					2B1FC										
	22																	
7	23		1B1DIRAC				2A1PEC											
	24								2C1FHS									
8	25		1B1DIRAC						2C3TCD									
	26						2A2CPEN											
	27								2C1FHS									
9	28			1C2NCIOP				2B2FIG										
	29							2B1FC										
	30						2A2CPEN						3D1BIPGN					
	31																	
	32		1B2FAI						2C3TCD									
10	33							2B1FC										
	34							2B1FC										
	35							2B1FC										
	36						2A2CPEN											
	37						2A1PEC											
11	38								2C1FHS									
	39	1A5CNR					2A2CPEN											
	40						2A1PEC											
12	41		1B3RP				2A2CPEN											
	42		1B3RP				2A2CPEN											
	43		1B1DIRAC															
13	44	1A3I					2A1PEC											
	45			1C2NCIOP			2A1PEC											
14	46		1B3RP					2B2FIG										
	47	1A1AV					2A2CPEN											
	48						2A2CPEN											
	49					1E1EITD												
