# Supplementary material for: Focus group on conflict management in the classroom in Secondary Education in Costa Rica: mixed methods approach
Source: Front Psychol. 2024 Oct 3;15:1407433. doi: 10.3389/fpsyg.2024.1407433 (PMC11483860; doi:10.3389/fpsyg.2024.1407433)
Supplement: Supplementary file 7 [file Data_Sheet_3.pdf]

Supplemenrary material
Question 3 Coded answers

		1. Tipology of the conflict					2. Aspects that affect the emergence of the conflict			3. Coping strategy					4. Teaching skills to manage conflict			
Participant	Textual Units	A. Interpersonal	B. Structural	C. Interests	D. Value	E. Information	A. Individual	B. Group	C. Social	A. Integration	B. Domination	C. Servility	D. Evitation	E. Commitment	A. Social	B. Emotional for conflict negotiation	C. Cognitive	D. Comunication
1	1																	
	2																	
	3								2C3TCD									
	4													3E1APBSI			4C1APCC	
2	5						2A2CPEN						3D1BIPGN					
	6													3E1APBSI				4D3CI
	7													3E1APBSI		4B1RE		
3	8													3E1APBSI				4D2UTVA
	9																	4D2UTVA
	10																	
4	11			1C4NI										3E1APBSI	4A2CPS			
	12													3E1APBSI	4A1EPC			
	13			1C4NI														
	14									3A1PSFAPC							4C1APCC	
5	15	1A4ICR							2C1FHS									
	16																	
	17									3A1PSFAPC					4A2CPS			
	18									3A1PSFAPC					4A2CPS			
	19																	
	20													3E1APBSI		4B3DPEAC		
6	21										3B1SIP							
	22						2A2CPEN							3E1APBSI	4A2CPS			
	23							2B1FC										
7	24													3E1APBSI		4B4FCSC		
	25								2C1FHS									
	26																	
	27									3A1PSFAPC					4A1EPC			
	28									3A1PSFAPC						4B4FCSC		
	29																	
8	30								2C1FHS	3A1PSFAPC					4A2CPS			
	31											3C1UPCO					4C1APCC	
	32												3D1BIPGN			4B2TPC		
	33									3A1PSFAPC						4B4FCSC		
9	34													3E1APBSI	4A1EPC			
	35										3B1SIP							
	36										3B1SIP					4B1RE		
10	37										3B1SIP							
	38									3A1PSFAPC					4A2CPS			
	39								2C1FHS				3D1BIPGN					
	40								2C1FHS									
	41								2C1FHS									
11	42										3B1SIP							
	43									3A1PSFAPC								
	44												3D1BIPGN					
	45												3D1BIPGN					
12	46		1B2FAI										3D1BIPGN					
	47																	
	48												3D1BIPGN					
	49																	
	50		1B2FAI															
	51					1E1EITD							3D1BIPGN					
13	52												3D1BIPGN					
	53		1B2FAI										3D1BIPGN					
14	54												3D1BIPGN					
	55																	
	56												3D1BIPGN					
	57																	
	58																	
	59		1B2FAI										3D1BIPGN					
	60																	
